# Theta entrainment of gamma modules: effects of heterogeneity and non-stationarity

**DOI:** 10.1186/1471-2202-13-S1-P170

**Published:** 2012-07-16

**Authors:** Ruben A Tikidji-Hamburyan, Carmen C Canavier

**Affiliations:** 1 Department of Cell Biology and Anatomy, Louisiana State University, New Orleans, Louisiana 70005, USA; 2 A.B. Research Institute for Nurocybernatics, Southern Federal University, Rostov-on-Don, 344090, Russia

## 

Transient synchronization of neurons in the gamma range (30-100 Hz) is believed to play an important role in attention, memory tasks[1] and other cognitive functions[2]. In many brain regions, gamma oscillations are modulated by the lower frequency theta rhythm[3]. There are at least two open questions with respect to gamma frequency synchronization: how this synchronization might occur between distal regions without direct connections and whether inputs from slow theta oscillations can synchronize populations of neurons firing at gamma frequency. It is well known that a low frequency input is capable of phase-locking a higher frequency oscillator, thus a population of gamma neurons with similar frequencies may synchronize due to common theta input.

Here we exploit maps based upon the phase resetting curves [4] (PRCs) to examine minimal model of a feed-forward network of oscillating neurons and to derive existence and stability criteria for phase-locking of high frequency oscillators by a common theta input. Then we extend these results for heterogeneous network with Gaussian-distributed periods and show good agreement among direct simulation of 200,000 Morris-Lecar model neurons, results obtained by PRC mapping of the same population, and theoretical predictions. These results may be extended to a special case when biological noise is approximated by treating the period of each gamma oscillator as non-stationary, specifically as an Ornstein-Uhlenbeck process (OUP) with a fixed time constant and variance. Good agreement between theory and the PRC mapping results were obtained for small variance and a sufficiently large time constant of the OUP such that the time scale is slower than the rate of convergence of gamma neurons to their preferred phase. However, the limitation of the method is that the predicted and actual distributions diverge with increasing OUP variance as well as with decreasing time constant. For example, if the PRC has two stable branches and the period varies rapidly compared to the speed of attraction of the stable locking point, phases corresponding to the unstable branches will have an inflated probability density compared to our theoretical predictions due to transient crossing of these phases as the fixed point switches between branches.

Finally we adopt this theory for the case in which there are multiple theta inputs to the gamma population and show that regions of existence and stability can thereby be significantly extended. Moreover, this result suggests a possible control scheme for switching the activity of a gamma population between synchrony, a cluster solution or a completely desynchronized pattern by varying the phase between theta inputs, which could theoretically lead to theta – gamma nesting, which is widely observed experimentally.
